# Incidental Renal Cell Carcinoma in Pelvic Malignancies

**DOI:** 10.7759/cureus.3829

**Published:** 2019-01-04

**Authors:** Fatima Shaukat, Muhammad Atif Mansha, Nasir Ali, Agha Muhammad Hammad Khan, Ahmed Nadeem N Abbasi

**Affiliations:** 1 Oncology, Agha Khan University, Karachi, PAK; 2 Oncology, Aga Khan University, Karachi, PAK

**Keywords:** renal cell carcinoma, dual malignancy, pelvic malignancy

## Abstract

Simultaneous diagnosis of renal cell carcinoma with pelvic malignancies is rare but a well-documented phenomenon. It is not uncommon to have incidental renal masses on imaging done for investigating other tumors. There are no established guidelines for the treatment of patients with dual malignancies. The management of such patients is challenging and requires a multidisciplinary approach.

We present a series of three cases with a diagnosed pelvic malignancy but further workup revealed a kidney tumor. Both the malignancies were evaluated individually and diagnosed as two different primary neoplastic lesions. This case series examines each distinct patient’s presentation, discusses the diagnosis, and compares and contrasts the findings while discussing the literature on this topic.

## Introduction

Renal cell carcinoma (RCC) is the most common malignancy of kidney. Its incidence is rising and almost doubled in past two decades in North America [1]. It is a well-known fact that RCC is associated with other primary malignancies such as bladder cancer, rectal cancer and non-Hodgkin's lymphoma but its association with pelvic tumors such as prostrate, cervix and endometrium is rare [2].

The optimal treatment strategies of such scenarios are not obvious in the literature [3]. Furthermore, diagnosing renal masses as a separate primary neoplasm and distinguishing them from metastases poses a challenge for the clinicians. The purpose of this report is to describe three distinctive patients who presented with pelvic malignancies and on further clinical assessment were found to have primary renal masses.

## Case presentation

Case 1

A 63-year-old male, a medical doctor in rural health care center, with known comorbidities of hypertension and type II diabetes mellitus, presented in urology clinic with complaints of increased frequency of urination for the past two years. There was no associated pain, blood, dribbling or hesitancy. On review of systems, he was found to have blurred vision in both eyes. His past medical and surgical histories were not significant. Although his family history was positive for diabetes mellitus and coronary artery disease in siblings, but there were no malignancies. His medications included metformin, acetylsalicylic acid, carvedilol, amlodipine and atorvastatin. He denied smoking, drinking alcohol or any other addiction. On general physical examination, he was anemic. Central nervous system examination was within normal limits. On chest auscultation, there were no added sounds. Abdomen was soft, non-tender with no hepatosplenomegaly on palpation. Upon digital rectal examination, prostate gland was enlarged, nodular and firm to hard in consistency.

**Figure 1:**
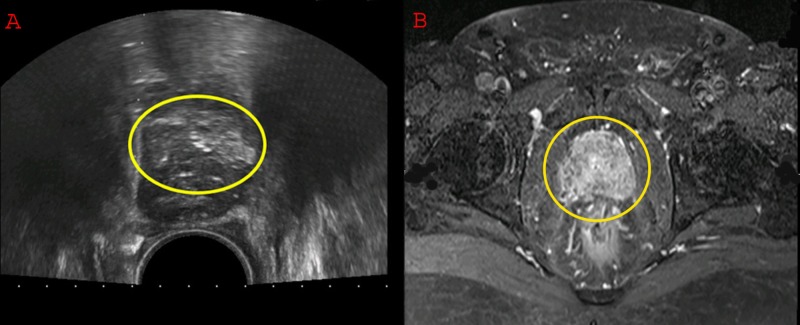
(A) Ultrasound of the pelvis showing an enlarged prostrate. (B) Magnetic resonance imaging (MRI) of the pelvis with contrast showing an enhancing lesion in the prostrate.

Suspecting a primary prostate disease, a serum prostate-specific antigen level was advised, which reported as 44.53 ng/ml. A transrectal ultrasonography-guided 12 core biopsy of prostate gland was planned which showed adenocarcinoma of prostrate. All the cores were involved by the disease with a Gleason score of 8. For staging purposes, he was further investigated with a magnetic resonance imaging (MRI) of the pelvis and a whole-body skeletal scintigraphy. On MRI prostate appeared heterogeneous and enlarged measuring 48 x 41 x 38 mm in anteroposterior, transverse and craniocaudal dimensions. Signal abnormality was seen in the peripheral zone on the left side representing a neoplastic lesion, infiltrating into the adjacent fat. Seminal vesicle on the right side was also involved; however, there were no enlarged lymph nodes (Figure 1A, 1B). Whole-body skeletal scintigraphy was negative for bony metastasis.

**Figure 2:**
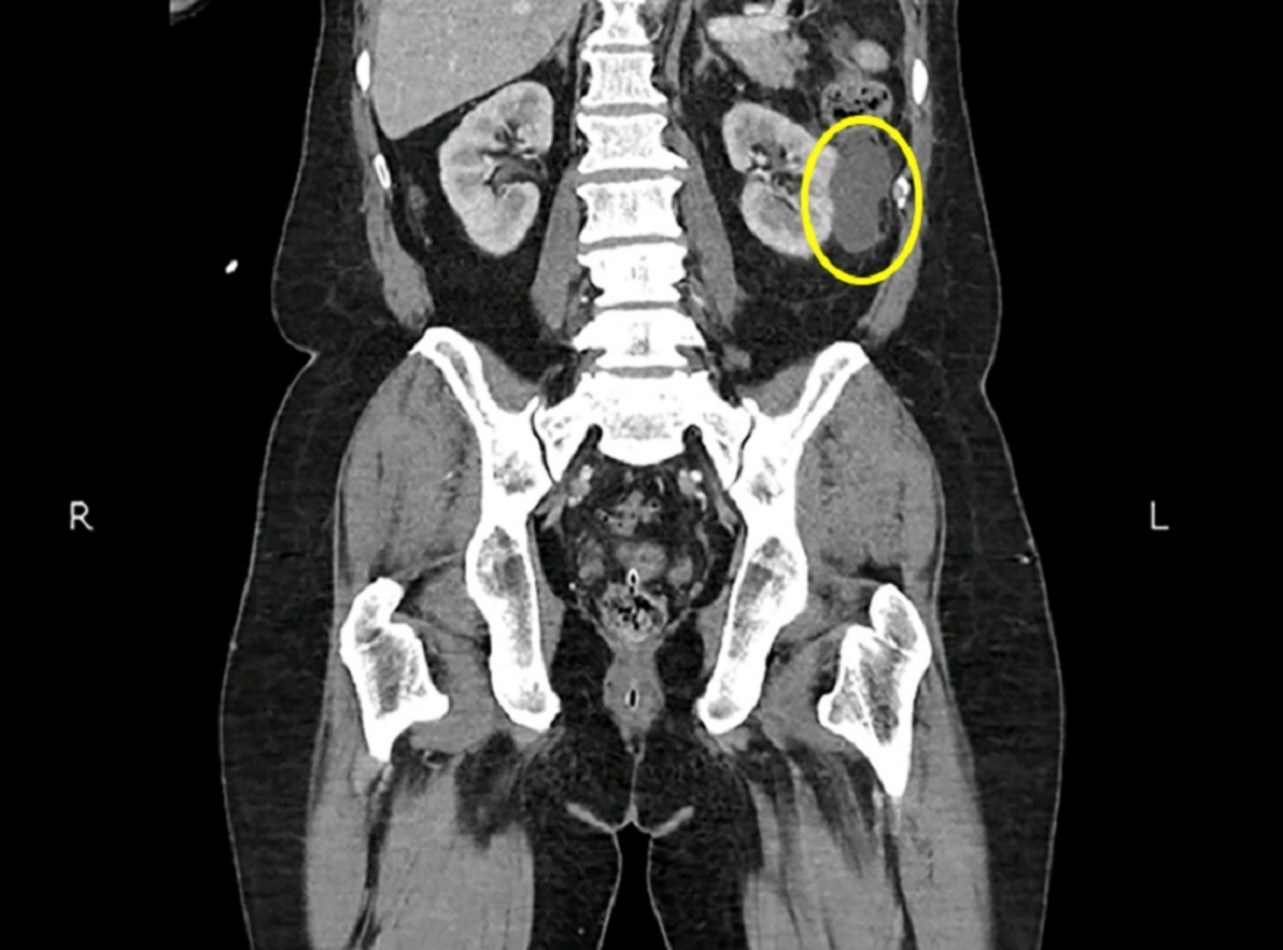
Computed tomography (CT) scan of the abdomen showing a heterogeneous lesion in the left kidney.

On the basis of the clinical findings, he was assigned a very high-risk group as per the prostate cancer risk group’s stratification. He was offered curative treatment with external beam radiation therapy to pelvis along with hormonal therapy. A computed tomography (CT) scan of abdomen and pelvis with intravenous contrast was done for radiation planning purposes. This CT revealed an incidental renal mass with enlarged paraaortic nodes (Figure 2).

Ultrasonography of abdomen complimented these findings. He underwent left paraaortic lymph node biopsy which showed small clusters of atypical epithelial cell, most likely renal origin with positive IHC – CKAE1/AE3, CD10 and vimentin focally positive. CT chest with intravenous contrast was done to complete the staging workup and that was normal.

He underwent left partial nephrectomy with pelvic and paraaortic lymph node dissection. Histopathology revealed papillary Grade 3 RCC with tumor confined to kidney only. Both perinephric resection margin and renal parenchymal margins were tumor free and lymphovascular invasion was not identified. A total of nine lymph nodes were removed and they all turned out to be positive for renal cell carcinoma. Pathological staging was pT1N1. For prostrate carcinoma external beam radiotherapy was delivered with intensity modulated radiation therapy (IMRT) technique delivering a dose of 7560 cGy in 42 fractions with radical intent along with androgen deprivation therapy (ADT).

Case 2

A 40-year-old female with a known case of hypertension presented in the gynecology oncology clinic with complaints of intermenstrual bleeding and increased urinary frequency for the last three months. The patient denied any significant medical or surgical history. She had no substantial family history. Her systemic examination was unremarkable. However, on her vaginal examination with Cusco’s speculum, a barrel-shaped cervix was visible with a lobulated mass in the left vaginal fornix. On palpation, the mass was firm in consistency, approximately 5 x 5 cm in size and there was no bleeding. Subsequently, a digital rectal examination was also performed which revealed a mass fixed to left pelvic side wall.

**Figure 3:**
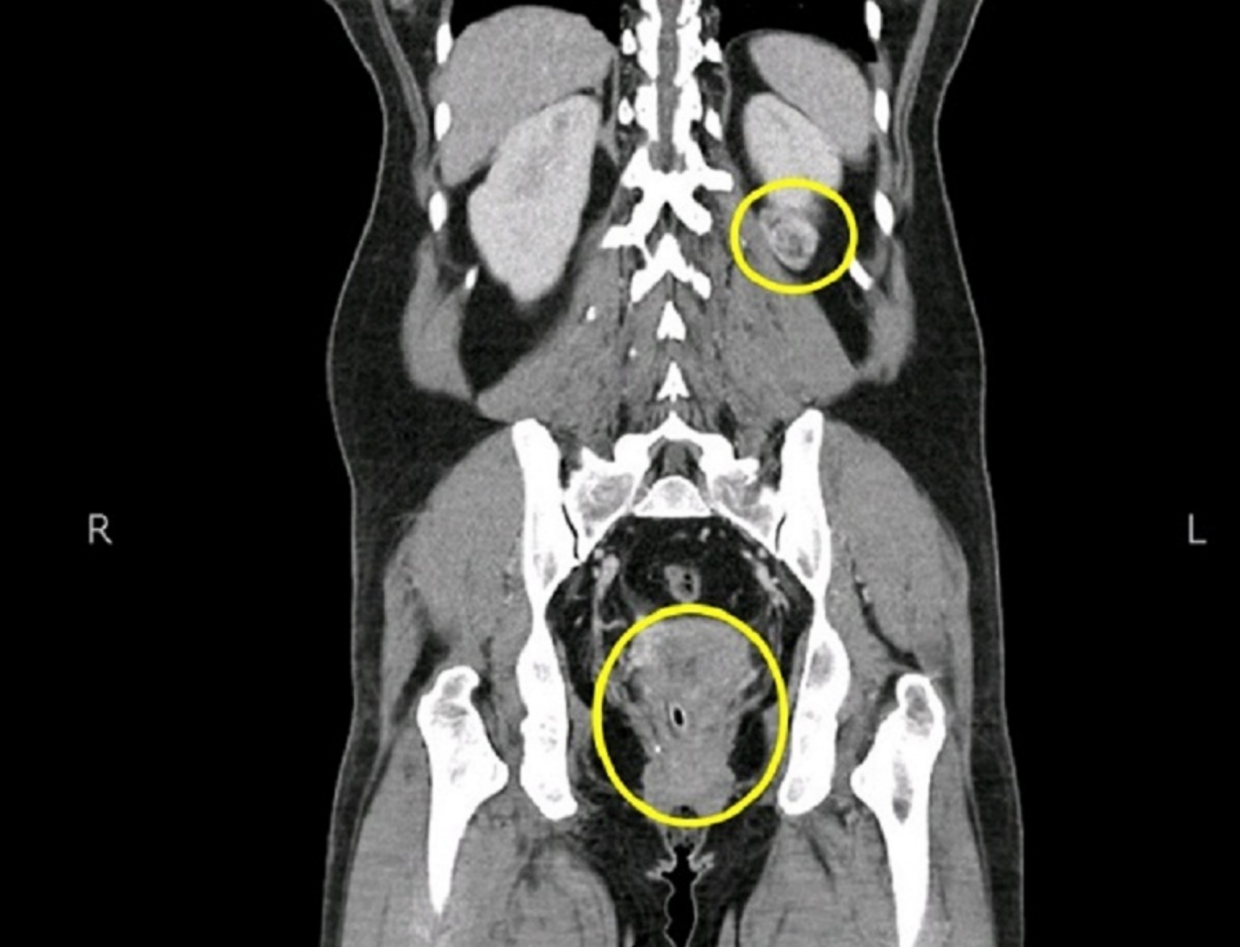
Computed tomography (CT) scan of the abdomen and pelvis showing a heterogeneous lesion in the cervix and the lower pole of the left kidney.

On investigating the mass her examination under anaesthetic (EUA) was done and biopsy was taken from the cervical mass which turned out to be non-keratinizing squamous cell carcinoma. CT abdomen and pelvis with intravenous contrast was performed which revealed enhancing lesion in cervix which is extending into the posterior parametria. Another positive finding was exophytic heterogeneous lesion arising from lower pole of left kidney, which was reported as primary renal neoplasm with abdominal pelvic lymphadenopathy (Figure 3).

The patient was referred to a urologist and the case was discussed in multidisciplinary tumor board and the consensus was made to manage the cervical cancer first due to its natural history followed by partial nephrectomy for left renal mass. For cervical carcinoma, she was treated with external beam radiation therapy with curative intent in definitive setting with a total dose of 5040 cGy in 28 fraction @ 1.8 Gy per fraction along with weekly concomitant cisplatin 40 mg/m^2^. She further received 24 Gy via tandem and ovoid brachytherapy in three fractions. She completed the treatment and tolerated the procedure well with limited pelvic and gastrointestinal (GI) toxicities. She is now planned for partial nephrectomy for her renal cell carcinoma.

Case 3

This is a 59-year-old, nulliparous, post-menopausal woman who was referred to gynecology oncology clinic by a local gynecologist. In recent past, she had complaints of vaginal spotting for two months for which she underwent total abdominal hysterectomy and bilateral salpingo-oophorectomy without any pre-operative investigations. Her remote past surgical history was significant for appendectomy in 2012 and tonsillectomy in 2014. Family history was significant for malignancy in her younger brother who had salivary gland carcinoma. Her gynecological examination revealed small induration at the anterior wall of vagina near the vault. Rest of the systemic examination was unremarkable.

The histopathology was reported as moderately differentiated endometrial adenocarcinoma, Grade 2; the lesion was invading more than 50% of myometrium. Size of the tumor was 4.5 x 3 x 1 cm in anteroposterior, transverse and craniocaudal dimensions. A positron emission tomography (PET) scan showed hyper metabolic soft tissue lesion involving vaginal stump extending into left adnexa with standardized uptake value (SUV) 16.4. This was most likely post-surgical changing and there was no evidence of distant metastasis.

**Figure 4:**
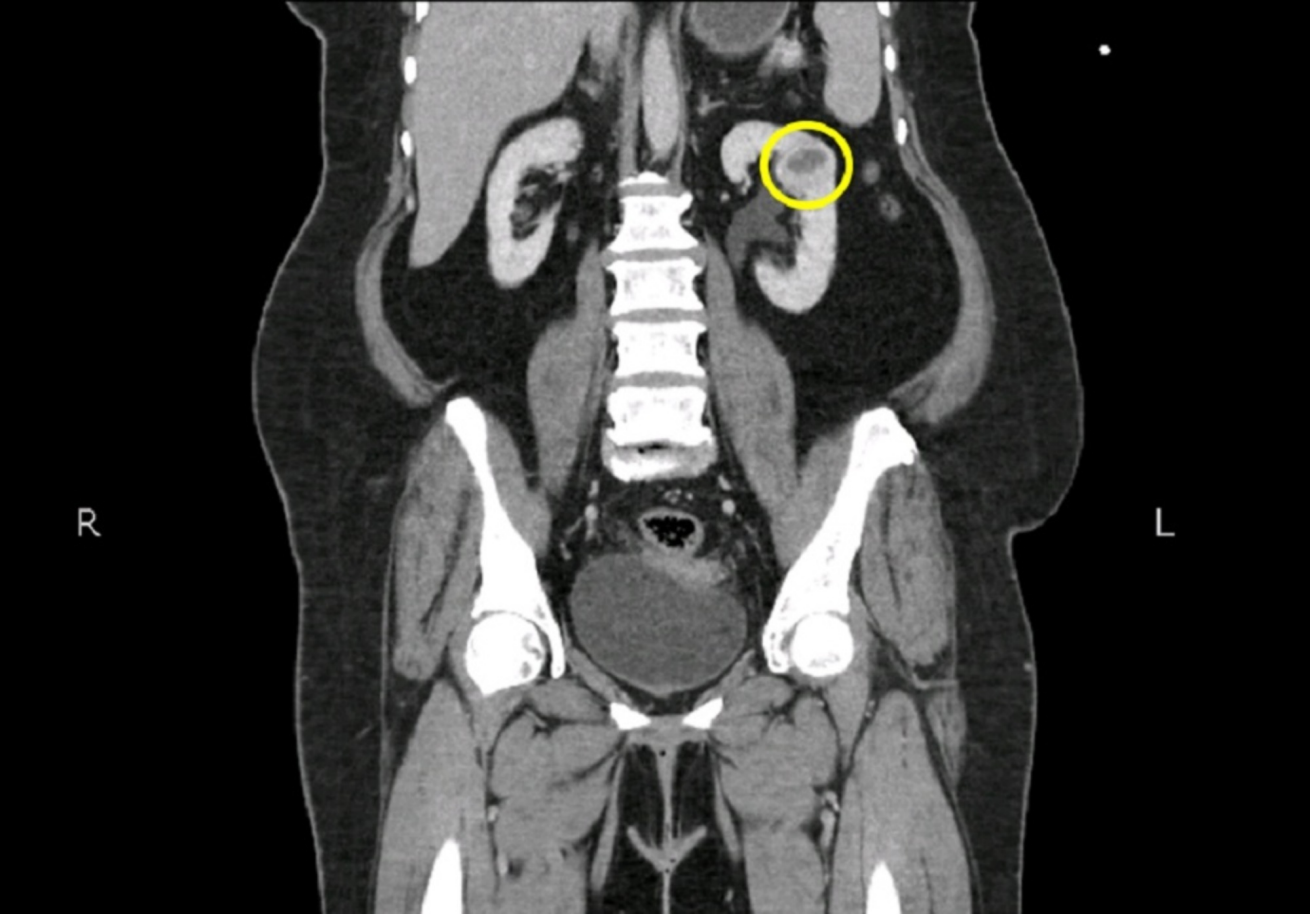
Computed tomography (CT) scan of the abdomen and pelvis (coronal view) showing a small lesion in the left kidney.

She was planned for adjuvant radiotherapy for which she underwent planning CT scan of abdomen and pelvis with intravenous contrast which revealed supplementary mass in left kidney (Figure 4). The mass was reported as renal cell carcinoma on radiology which was further confirmed on ultrasound abdomen.

The case was discussed in urology multidisciplinary team (MDT) and consensus was made to perform ultrasound-guided biopsy of the renal mass. The patient, however, refused to undergo the procedure so she is now planned for radiotherapy to the whole pelvis with three-dimensional conformal radiotherapy (3DCRT) delivering the total dose of 4500 cGy @ 1.8 Gy per fraction followed by two fractions of brachytherapy one week apart.

## Discussion

The incidence of multiple primary malignancies has been long reported in the literature [1]. Warren and Gates were the first ones who described multiple malignant tumors as a separate entity [4]. Synchronous malignancies are defined as those diagnosed concurrently or within three months of the diagnosis of former [5].

RCC accounts for more than 90% of cancers arising from the kidney [6]. The risk of developing antecedent, synchronous and metachronous primary malignancies is high in patients diagnosed with RCC. Cancer in the prostate, bladder, lung, breast, colon and rectal cancer, malignant melanomas (MM) and non-Hodgkin's lymphomas (NHL) are the most common malignancies associated with RCC [2]. Arjunan et al. reported a rare case of synchronous presentation of carcinoma breast with RCC [7].

In contrary to this, sometimes RCC remains silent and is discovered when patients are being investigated for other primary malignancies. Piccinini et al. have reported six cases of RCC occasionally diagnosed during initial staging workup of a rhino pharyngeal carcinoma, gastric cancer, Waldenström's disease, NHL, and breast cancer [3].

Dafashy et al. reported a series of three cases in which each patient was diagnosed with RCC and a unique synchronous gastrointestinal malignancy, i.e., a carcinoid tumor of the small bowel, a mucinous appendiceal neoplasm and hereditary nonpolyposis colorectal cancer [8]. In addition to this, a team of surgeons also shared their experience of laparoscopic removal of RCC and sigmoid colon cancer simultaneously [9-11]. Another case report has also highlighted the management of concurrent RCC and pancreatic ductal adenocarcinoma [12, 13].

Synchronous association of RCC with hematological malignancies has also been published. A study conducted at Memorial Sloan Kettering Cancer Center identified 15 patients who had both RCC and malignant lymphoma at some point in time in their lives. Twelve of these patients had synchronous malignancies [14, 15]. Similarly, Ozturk et al. described the coexistence of RCC with multiple myeloma in two patients [16]. Another paper is published relating RCC with breast carcinoma [17-19]. Multidisciplinary approach plays a vital role in the management of such patients.

Our patients were booked for radiation therapy to pelvis. It is our usual practice to acquire images of abdomen along with pelvis in radiation planning CT scan to include the opaque markers which we place for patient alignment. Incidentally, all of them were found to have a renal mass. These scans were discussed with the radiologist and a clinical diagnosis of RCC was suggested.

In all the cases both malignancies were treated with curative intent. Another common finding among all three cases was that they were diagnosed on CT scan as an incidental finding. This reflects the widespread use of diagnostic imaging as standard of care utilized when assessing the patients with pelvic malignancies.

## Conclusions

All cancer patients should be evaluated thoroughly and discussed in multidisciplinary tumor board before starting any intervention. Diagnostic imaging should be taken as an important modality in investigations as advancement in radiology has increased the incidence of detection of unexpected lesions. Each lesion site should be diagnosed and treated as a separate entity.
